# The Symptoms and Probable Causes of Hæmoptysis, as It Prevailed in Little Rock, Arkansas

**Published:** 1857-08

**Authors:** C. McAlmont

**Affiliations:** Charleston, Ill.


THE NORTH-WESTERN

### MEDICAL AND SURGICAL JOURNAL.

(NEW SERIES.)

VOL. VI. AUGUST, 1857. NO. 8.

### ORIGINAL COMMUNICATIONS.

ARTICLE I.—*The Symptoms and Probable Causes of Hœmoptysis as it prevailed in Little Rock, Arkansas, last December, January and February, and the Treatment.* Read to the Esculapian Society, 28th May, 1857, by DR. C. MCALMONT.

There occurred about forty or fifty cases of this disease, at the time mentioned, and it attacked persons of the most opposite degrees of health and constitution, but generally those of feeble constitution, either natural or induced.

This disease is not of common occurrence in this section of country. Chronic and tubercular affections of the lungs are very rare, but acute inflammation of that organ prevails extensively through the winter months.

The symptoms preceding and following the attack were not uniform. In some cases it came on suddenly and without previous warning, but more generally it was preceded by cold extremities and chilly sensations, loss of appetite, headache, and slight debility, with feelings of oppression. These symptoms were followed by some fever, and were either remittent or intermittent in character, lasting from one to six or eight days before the hemorrhage took place. The hemorrhage was attended and preceded by cold extremities and chilly sensations; the pulse feeble and small, beating from ninety to one hundred strokes per minute. As the hemorrhage advanced, and in proportion to the quantity of blood lost, the pulse sank and became more rapid and feeble, the body growing cold and covered with perspiration.

These symptoms lasted from one to twelve hours, according to the treatment and severity of the attack.

The first indications of improvement were increased strength and fullness of the pulse, followed by warmth of body and extremities, the hemorrhage growing less until it had entirely ceased, or nearly so. The reaction, in some cases, was very violent, but generally feeble. As the symptoms preceding the hemorrhage were remittent or intermittent, so was the hemorrhage with its attending symptoms, and you might look for a recurrence of the hemorrhage in one, two or three days, if intermittent, unless cut short by treatment, and from four to six hours, if remittent, the hemorrhage never ceasing entirely until there had been several exacerbations and remissions.

The hemorrhage was of a passive character, but sometimes it continued after reaction had fully taken place, and became active. This only occurred in two or three cases. The prostration following these hemorrhages was not, in general, as great as might have been expected, from the quantity of blood lost, which was great in some cases, but more often the quantity was small, ranging from two to sixteen ounces. The tongue was always more or less furred, sometimes white; more often yellow or brown. The bowels were generally costive, and showing a want of bilious secretion; the countenance *sallow;* eyes dull and surrounded with a blue circle, and, in some cases, became tinged with bile. The thirst was very great, and frequently attended with nausea and vomiting; the blood was florid and frothy; the cough attending these attacks was sometimes considerable, at other times slight, accompanied with more or less pain and tickling sensations; auscultation revealed sibilant and sonorous rattles, when the blood was in the air passages, and at points where there was dullness on percussion (which only existed in a few cases), there was no respiratory murmur, and in its place a crepitous rattle.

PROBABLE CAUSES.

The predisposing causes may have been a feeble, frail constitution, debility from previous disease, narrow, contracted chest, diseased lungs, a hemorrhagic diathesis, and malaria, which seems to have been the most prominent.

The exciting causes may have been sudden transition from hot to cold, fatigue, mental excitement, indigestible food, or any other cause calculated to derange the natural and healthy functions of the body.

The proximate cause must have been congestion of the mucous membrane of the air passages, and, in the most violent cases, when there was dullness on percussion and crepitous rattle in auscultation, the congestion must have extended to the parenchyma, and the hemorrhage proceeding from the air cells, as well as the mucous membrane.

The prognosis was favorable, except in those cases where auscultation and percussion revealed abnormal sounds to a considerable extent, or previous disease existed.

The diseases most prevalent just previous to this time were dysentery, attended with more than usual hemorrhage; also there occurred, about this time, several cases of apoplexy, and a few cases of congestive, intermittent and remittent fever, and two of these cases were attended with alarming apistaxis.

Taking all these facts into consideration, it is very evident that there was a general tendency to congestion, followed by hemorrhage.

TREATMENT.

The treatment adopted and most successful was large doses of opium and quinine, from one to three grains of the former and five to twenty of the latter, repeated in four or eight hours if necessary. Strong revulsive remedies were applied to the chest, stomach, back and extremities; mustard plasters were generally used for this purpose; the bowels to be opened with calomel or the compound cathartic pill, the calomel to be continued in alternate doses, if indicated by want of bilious secretion and torpidity of the bowels. If the symptoms continued bad, blisters were applied to the chest and extremities. When the hemorrhage continued after reaction had fully taken place, and the pulse full and strong, venesection should be resorted to, with the internal use of acid drinks, ice and astringents. Acetate of lead is the best astringent, and was generally used, some preferring gallic acid.

The important question arises in the mind of every physician, why was congestion more frequently followed by hemorrhage in these cases, and at this time, than usual? I will not attempt to advance any theory, but merely state a few facts that may have some bearing on the question. The summer and fall were unusually hot, the thermometer ranging between ninety and one hundred day after day, and in houses not protected by shade, it rose to one hundred and six and eight. When winter set in it was unusually cold, and subject to frequent and sudden chages; the atmosphere was very damp and chilly; vegetables were very scarce and indifferent in quality, and the fruit was dwarfish and badly matured; consequently there was a greater consumption of meat than usual.

*Charleston, Ill, 28th May,* 1857.

